# Multiple brain metastases in a patient with ypT0N0 micropapillary urothelial carcinoma of the bladder

**DOI:** 10.1016/j.eucr.2021.101838

**Published:** 2021-09-13

**Authors:** Srikar Kuppa, Jeffrey Wei, Marshall Strother, Curtis Miyamoto, Michael Weaver, Alexander Kutikov

**Affiliations:** a Department of Urology, Temple University School of Medicine, Philadelphia, PA, USA; b Division of Urologic Oncology, Fox Chase Cancer Center, Philadelphia, PA, USA; c Department of Radiation Oncology, Temple University School of Medicine, Philadelphia, PA, USA; d Department of Neurosurgery, Temple University School of Medicine, Philadelphia, PA, USA

**Keywords:** Muscle invasive bladder cancer, Neoadjuvant chemotherapy, Brain metastass

## Abstract

Radical cystectomy (RC) after neoadjuvant chemotherapy (NAC) is the gold standard for management of muscle-invasive bladder cancer (MIBC). Patients without residual tumor at the time of extirpative surgery (ypT0) have excellent prognosis. Distant metastases in this population are rare. We present a unique case of a patient with ypT0N0 urothelial carcinoma (UC) with rapid development of metastasis to the brain.

## 1 Introduction

Neoadjuvant chemotherapy (NAC) followed by radical cystectomy (RC) is the standard of care for muscle-invasive bladder cancer (MIBC). In fact, some 30% of patients who undergo RC after NAC, do not harbor residual malignancy in the bladder (ypT0).^1^ These patients with ypT0 pathology exhibit superior survival compared to those with residual cancer.^2^ Additionally, lymph node recurrence and metachronous solid organ metastases in these patients are very uncommon.^2^ In this report, we present, to our knowledge, the first documented case of a patient with ypT0N0M0 UC who developed symptomatic brain metastases 8 months following RC.

## 2 Case presentation

A 58-year-old man with a 35 pack-year smoking history presented to his primary care physician with mild abdominal pain, dysuria, and urinary frequency. Urinalysis showed microhematuria and urine cytology and fluorescence in situ hybridization (FISH) testing suggested UC. Computed tomography urogram (CT) confirmed a bladder mass and metastatic workup was consolidated with negative chest imaging.

**Fig. 1:**
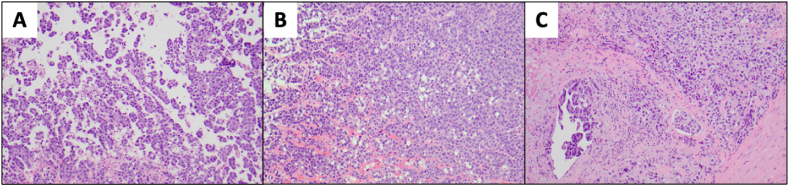
TURBT Specimen Histology. A) Urothelial carcinoma with Micropapillary Features, 100x. B) Urothelial Carcinoma with Micropapillary Features and Solid-Pattern Urothelial Carcinoma, 100x. C) Urothelial Carcinoma with Micropapillary Features and Lymphovascular Invasion, 100x.

The patient underwent transurethral resection of bladder tumor (TURBT) at an outside facility, which documented high grade muscle-invasive UC with lymphovascular invasion (LVI) and 20% micropapillary features (Fig. 1). The tumor was incompletely resected. Skull to thigh, florine-18 2-fluoro-2-deoxy-D-glucose-positron emission tomography/computed tomography (FDG-PET/CT) scan demonstrated uptake in the known bladder mass as well as two suspicious right pelvic lymph nodes. At an outside cancer center, the patient was started on a NAC regimen with six cycles of gemcitabine, cisplatin, and paclitaxel. Due to paclitaxel-induced neuropathy, the patient finished the last two cycles with gemcitabine and cisplatin alone. Two weeks after completing NAC, the patient developed a lower extremity deep venous thrombosis (DVT). He was started on apixaban and an inferior vena cava filter was placed. Seven months following his initial presentation, and 8 weeks after completing NAC, the patient underwent RC with a Studer-type ileal neobladder urinary diversion. The pelvic lymph node dissection was intentionally limited to calibrate perioperative risks in view of the patient's recent DVT. Pathologic examination of the bladder revealed no residual tumor, negative margins, and 8 negative lymph nodes, including those that revealed increased FDG uptake on pre-operative PET CT. Final staging was ypT0N0. Patient recovered well from surgery with excellent neobladder function. A 5-month post-operative CT of the chest and CT urogram demonstrated no evidence of disease.

**Fig. 2:**
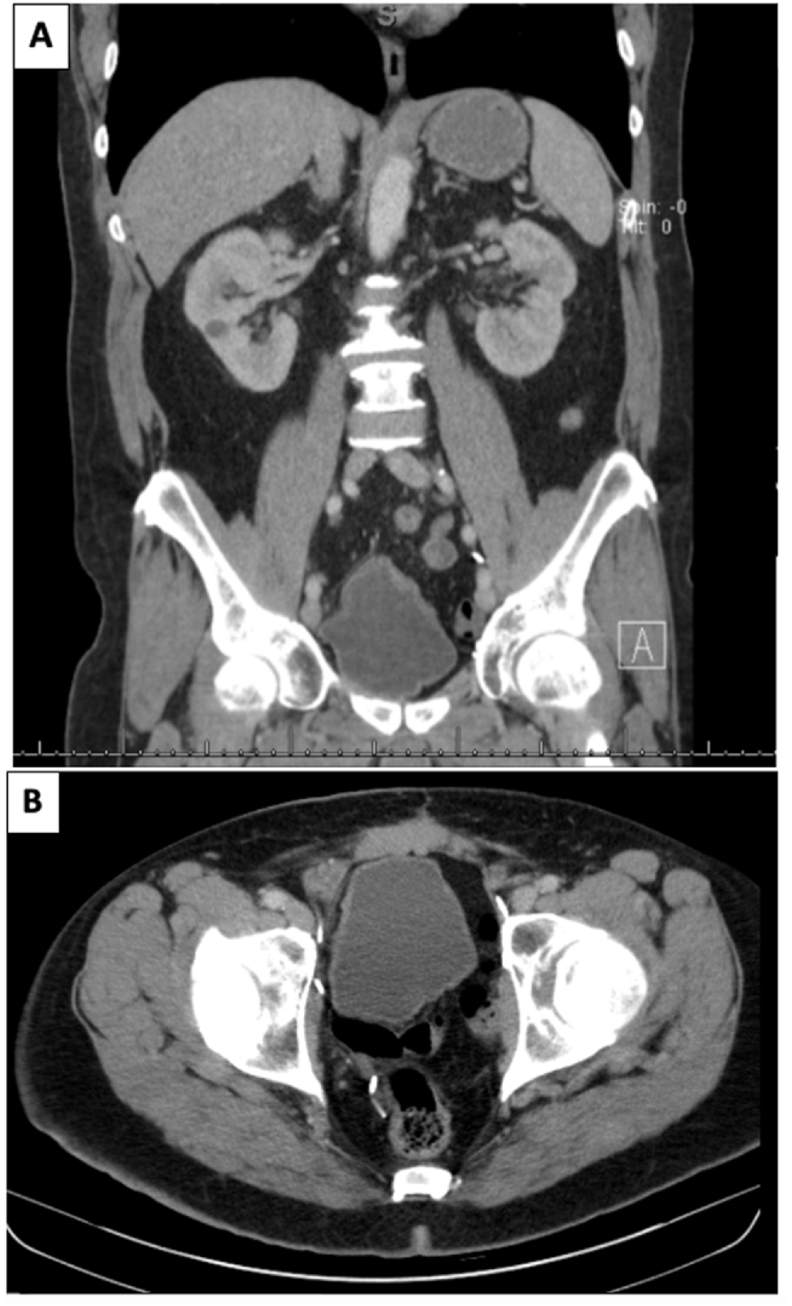
8-Months Post-Operative CT Abdomen and Pelvis with Contrast Demonstrating Intact Neobladder with no Evidence of Lymphadenopathy or Metastatic Disease. A) Coronal. B) Axial.

**Fig. 3:**
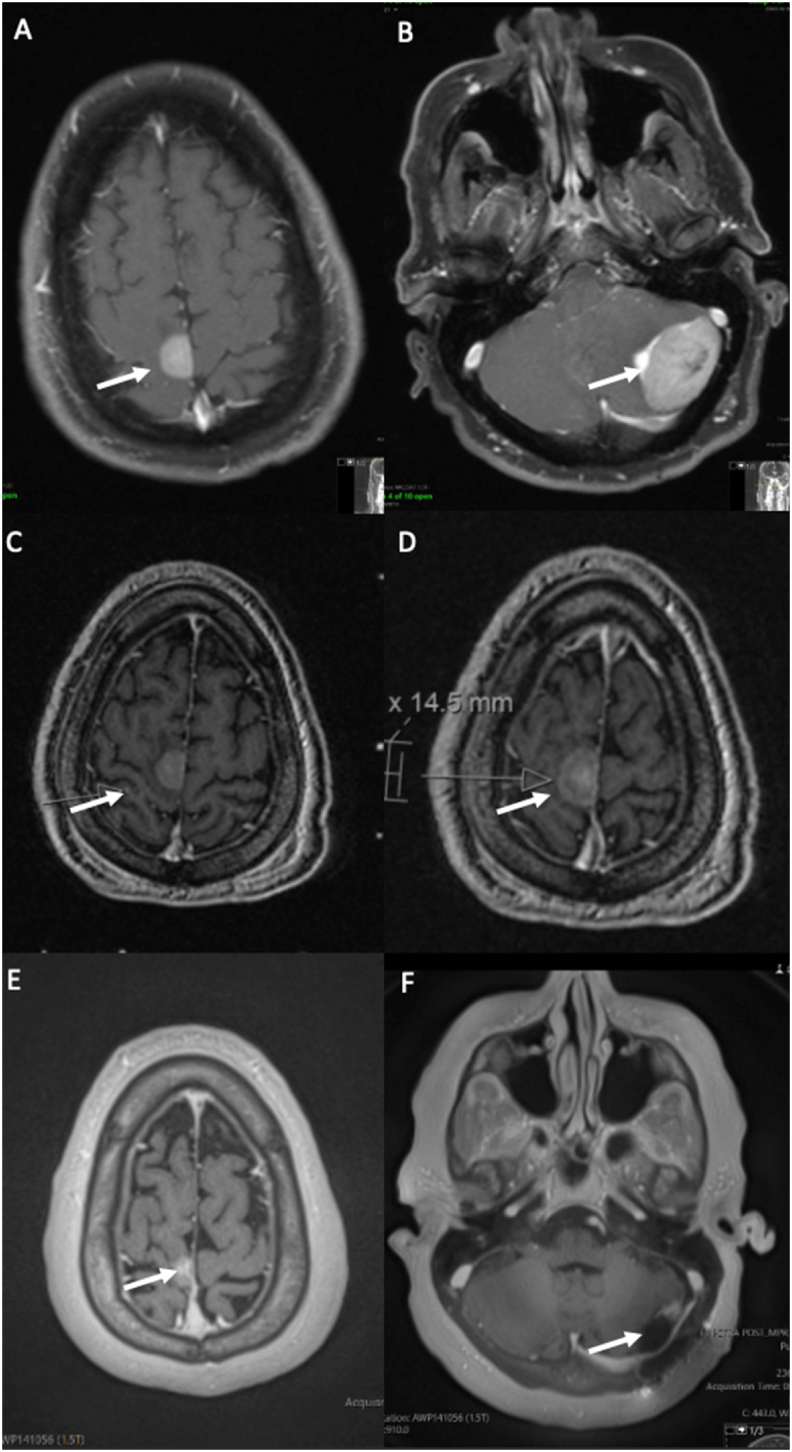
T1-weighted post-contrast brain MRI images. A) Lesion in right posterior frontal parasagittal cortex (arrow). B) Left cerebellar lesion (arrow) with marked vasogenic edema and compression of the fourth ventricle. C) Post-craniotomy imaging showing new punctate lesion (arrow). D) Post-craniotomy imaging showing increased size of right posterior frontal parasagittal cortex lesion (arrow). E) Post-craniotomy and radiotherapy imaging of right posterior frontal parasagittal cortex lesion site (arrow). F) Post-craniotomy and radiotherapy imaging of left cerebellar lesion site (arrow).

Approximately 8 months after RC, the patient presented with nausea, vomiting, dizziness, weakness and new onset of urinary incontinence. Repeat CT chest/abdomen/pelvis again showed intact neobladder with no evidence of lymphadenopathy or metastatic disease (Fig. 2). However, MRI of the brain found a large tumor in the left cerebellar hemisphere and a smaller lesion in the right posterior frontal parasagittal cortex (Fig. 3). The left cerebellar mass measured 4.9 × 3.4 × 2.6 cm with surrounding vasogenic edema resulting in mass effect compressing the ventricle. Due to the size and symptomatic nature of the cerebellar lesion, the patient underwent a left craniotomy with resection of the left cerebellar tumor, which revealed metastatic urothelial carcinoma with micropapillary features. Three weeks post-craniotomy, MRI of the brain showed a size increase in the parasagittal cortex lesion two new punctate lesions in the parasagittal right frontal lobe and the right parietal lobe which were suspicious for plural metastases. The patient received stereotactic radiosurgery (SRS) for the remaining lesions via gamma-knife and linac-based SRS. He recovered well post-craniotomy and his current treatment plan includes radiation therapy with immunotherapy and close radiographic surveillance. The patient is now 16 months post-RC with latest urology follow up at 15 months post-RC.

## 3 Discussion

While ypT0 stage is associated with excellent prognosis, with reported 5-year disease-free survival of 84–88%,^2^ this case represents an unexpected scenario for a patient with ypT0N0 UC following NAC and RC.^1^ Though uncommon, recurrence in ypT0 disease is usually due to regional lymph node spread and is seen in 3–7% of patients.^1^ Typical distant metastatic sites of UC include bone, lung, liver, and peritoneum.^1^ Our patient had no evidence of disease on routine surveillance at 5-months CT chest/abdomen/pelvis. The pelvic lymph nodes apparent on the initial PET-CT imaging were resected and there were no concerning findings of follow up scans. Patient's brain metastases were discovered when the patient became symptomatic.

Brain metastases in patients with bladder cancer are rare, and are both a diagnostic and therapeutic challenge. Description of brain metastases from UC are sparse, being found in an estimated 1–3% of metastatic UC patients.^3^ Moreover, the negative whole body FDG-PET/CT scan prior to RC could be explained by either a rapidly growing mass that developed following cystectomy or reflect physiologically-high FDG uptake by the brain masking an underlying metabolically active small metastatic lesion.^4^ Given rapid kinetics of new brain lesions development following resection, the former scenario is most likely.

Surgical intervention for bladder UC brain metastases is rarely reported since most patients present with concomitant extracranial disease. Fokas et al. reported that in a study of 62 patients with brain metastasis, metastasectomy with radiotherapy was not associated with a survival advantage over radiotherapy alone with a median overall survival of 9.6 vs. 8.9 months (p < 0.70).^3^ Resection in this case was undertaken for diagnostic purposes given that patient's pre-test probability for harboring intracranial metastases was low due to ypT0 pathology and lack of other metastatic sites.

Because clinical outcomes can vary in patients with ypT0 disease, this case demonstrates the importance of risk-stratifying such patients to inform clinicians’ suspicion of metastatic disease and the necessary level of surveillance.^5^ Kassouf et al. performed multivariate analysis of patients with ypT0 UC and found LVI in TURBT specimens predicted a significantly worse prognosis than TURBT specimens without LVI.^2^ Moreover, this patient had micropapillary features on histology for both the TURBT and craniotomy specimens. Like LVI, micropapillary features represent a highly aggressive UC subtype.^5^

## 4 Conclusion

Patients who undergo NAC and have no residual tumor upon RC (pT0) typically have superior oncologic outcomes. Our case report adds to the evolving literature on optimal management of such patients and reinforces the importance of future work in risk stratification and treatment of patients with variant histology.

## Support/financial disclosures

None.

## Declaration of competing interest

None.
